# Supplementary material for: Pan-active imidazolopiperazine antimalarials target the Plasmodium falciparum intracellular secretory pathway
Source: Nat Commun. 2020 Apr 14;11:1780. doi: 10.1038/s41467-020-15440-4 (PMC7156427; doi:10.1038/s41467-020-15440-4)
Supplement: Supplementary file 1 — Supplementary Information [file 41467_2020_15440_MOESM1_ESM.pdf]

SUPPLEMENTARY MATERIALS

Pan-active imidazolopiperazine antimalarials target the *Plasmodium falciparum* intracellular secretory pathway

LaMonte et al. 2020

- **Supplementary Figures 1-7**
- **Supplementary Tables 1-4**
- **Supplementary Methods (Conjugation of Coumarin-1 and NBD with GNF179)**
- **Supplementary References**

SUPPLEMENTARY FIGURES

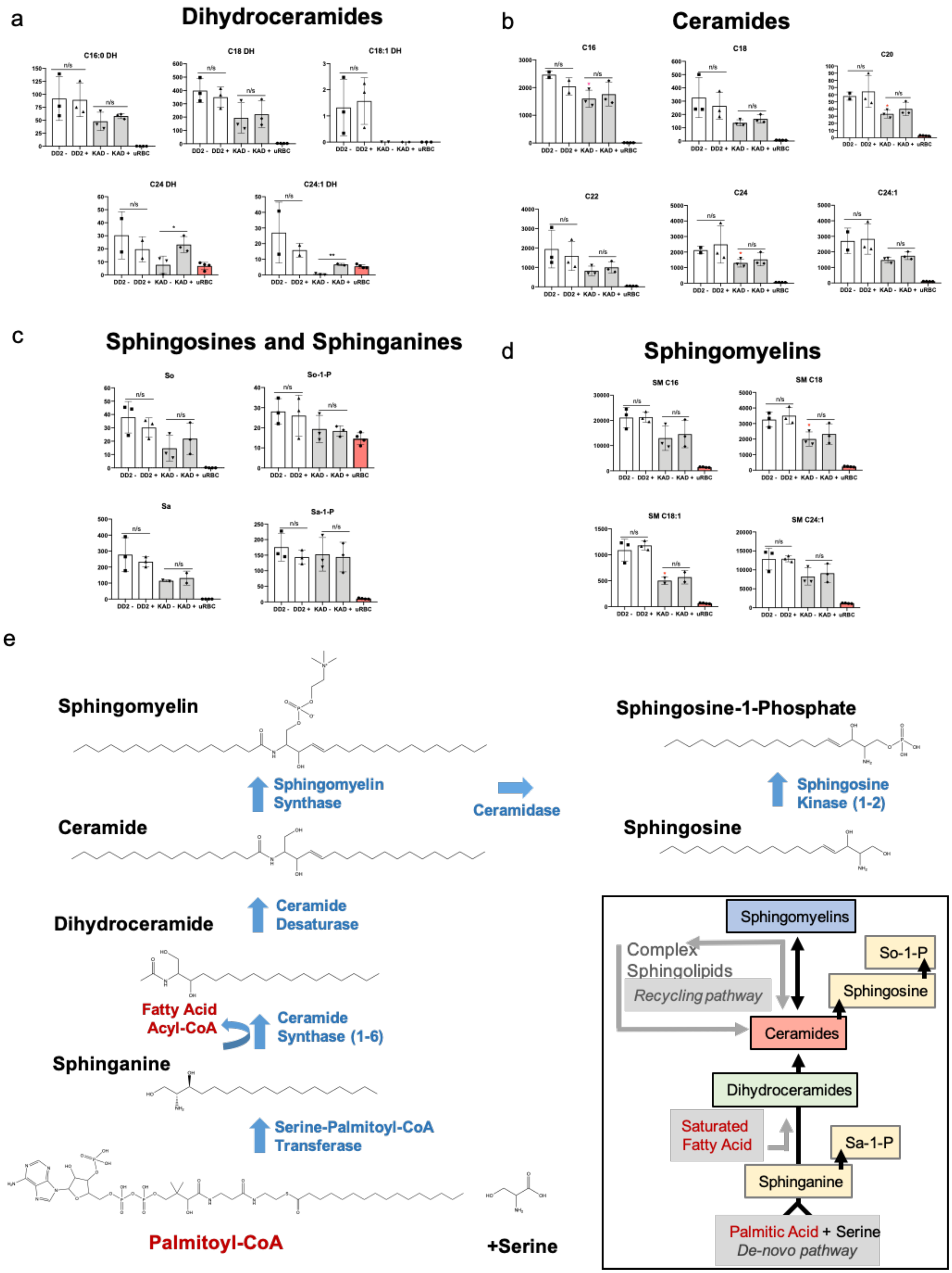

Supplementary Figure 1. Sphingolipid profile of isolated *P. falciparum*. Wild Type and PfCARL Mutant. Composite profile of 19 sphingolipids from untreated wild type Dd2 (DD2-) and *pfcarl* triple mutant (KAD-) as well as parasites treated with 25nM GNF179 (DD2+, KAD+). Lipids were normalized

to protein and reported as mean±SEM indicated by the error bars (n=3 independent experiments). An unpaired, two-tailed t-test was employed to compare between untreated and treated wild-type and mutant lines. Level of significance in the difference between treated vs untreated populations is indicated by solid black lines and a black *, while significance of untreated *pfcarl* mutants with respect to the untreated wild type populations is indicated by a red *, where * is $p < 0.05$, ** is $p < 0.01$, and *** is $p < 0.001$. Sphingolipid profile comprises (a) dihydroceramides (DH), (b) ceramides (C), (c) sphingosine (So), sphingosine-1-phosphate (So-1-P), sphinganine (Sa), sphinganine1-phosphate (Sa-1-P) and (d) sphingomyelins (SM) with varying fatty acid chains and degrees of saturation. Pink bars represent sphingolipid levels in uninfected red blood cells (uRBC). (e) Diagram of the sphingolipid synthesis and conversion pathway.

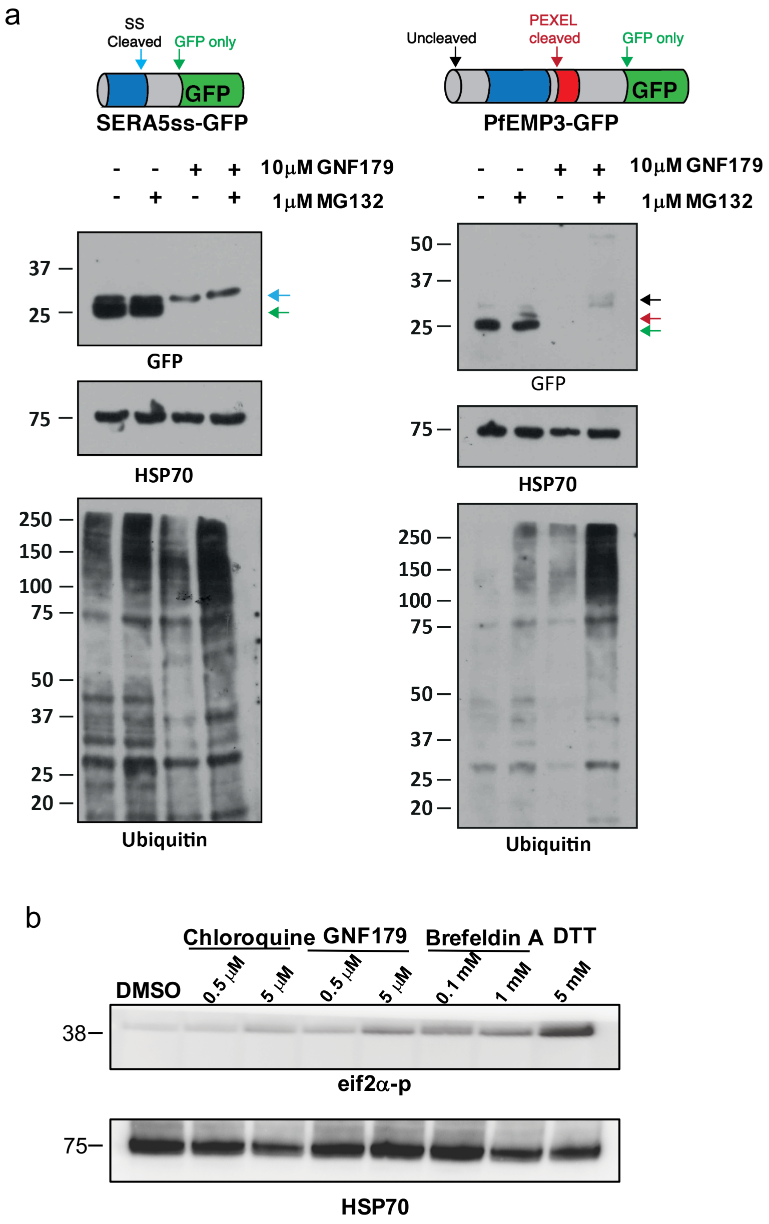

Supplementary Figure 2. (a) Western blots of *P. falciparum* strains expressing SERA5ss-GFP or PfEMP3-GFP treated with GNF179 and/or MG132 for 3 hours were probed with anti-GFP, anti-HSP70 and anti-Ubiquitin antibodies. For the secreted SERA5ss-GFP reporter, the blue arrow indicates the signal-sequence cleaved form and the green arrow indicates the GFP-only species. For the exported PfEMP3-GFP reporter, the black, red and green arrows indicate the full-length, PEXEL-cleaved and GFP only species respectively. (b) Western blots of *P. falciparum* Dd2 strain treated for 90 minutes with chloroquine, brefeldin A, GNF179 and dithiothreitol (DTT) were probed with anti-eIF2α-p and anti-HSP70 as loading control.

.

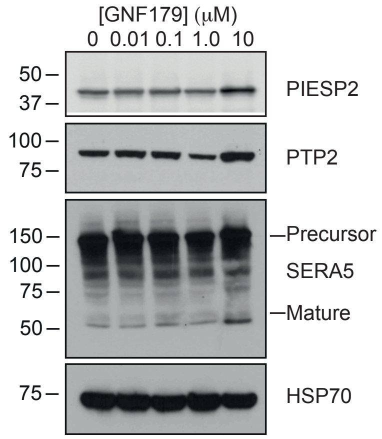

Supplementary Figure 3. Western blot showing effects by a 3-hour pulse of increasing concentrations of GNF179 on known ER-trafficked proteins in P. falciparum. PIESP2 and PTP2 are examples of PEXEL-containing exported proteins. SERA5 is a secreted protein while HSP70 is a cytosolically expressed protein that lacks a signal sequence required for ER entry.

.

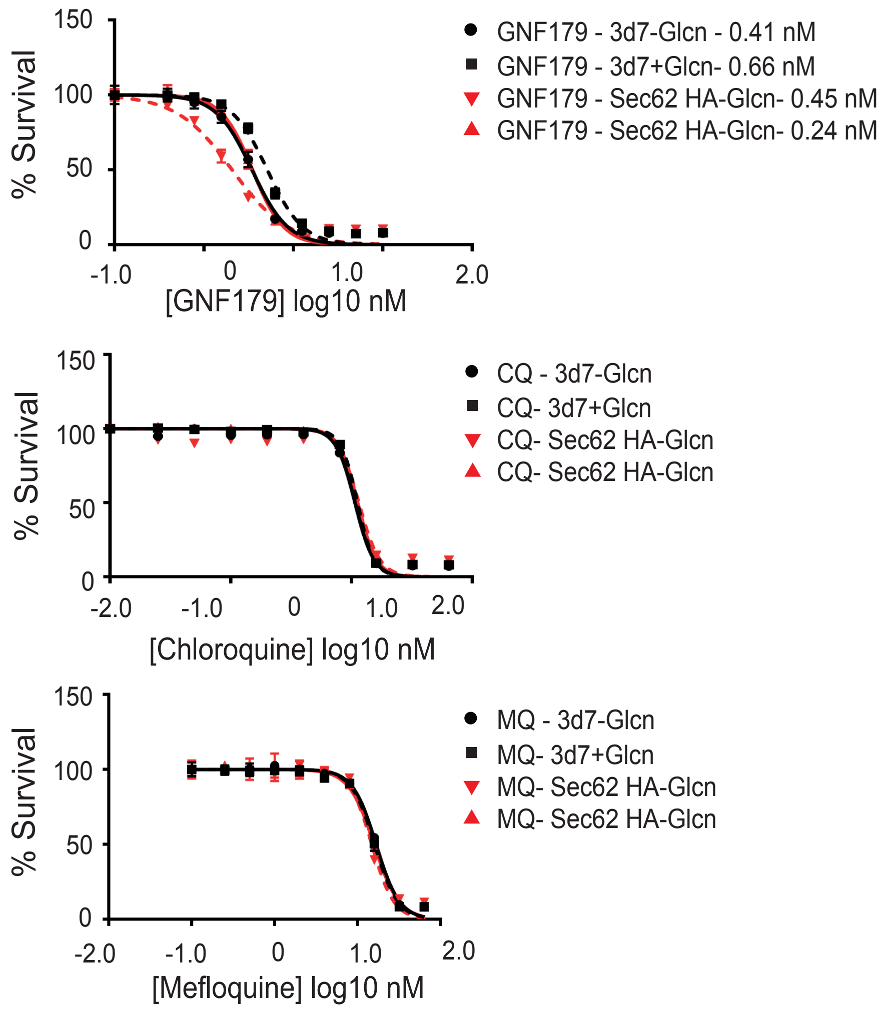

Supplementary Figure 4. Dose response curves for GNF179 in the SEC62 knockdown parasites, compared to wildtype 3D7 parasites, with and without the addition of N-acetyl Glucosamine (Glcn) for the 3 compounds indicated, GNF179, Chloroquine (CQ) and Mefloquine (MQ). Values are for three independent replicates.

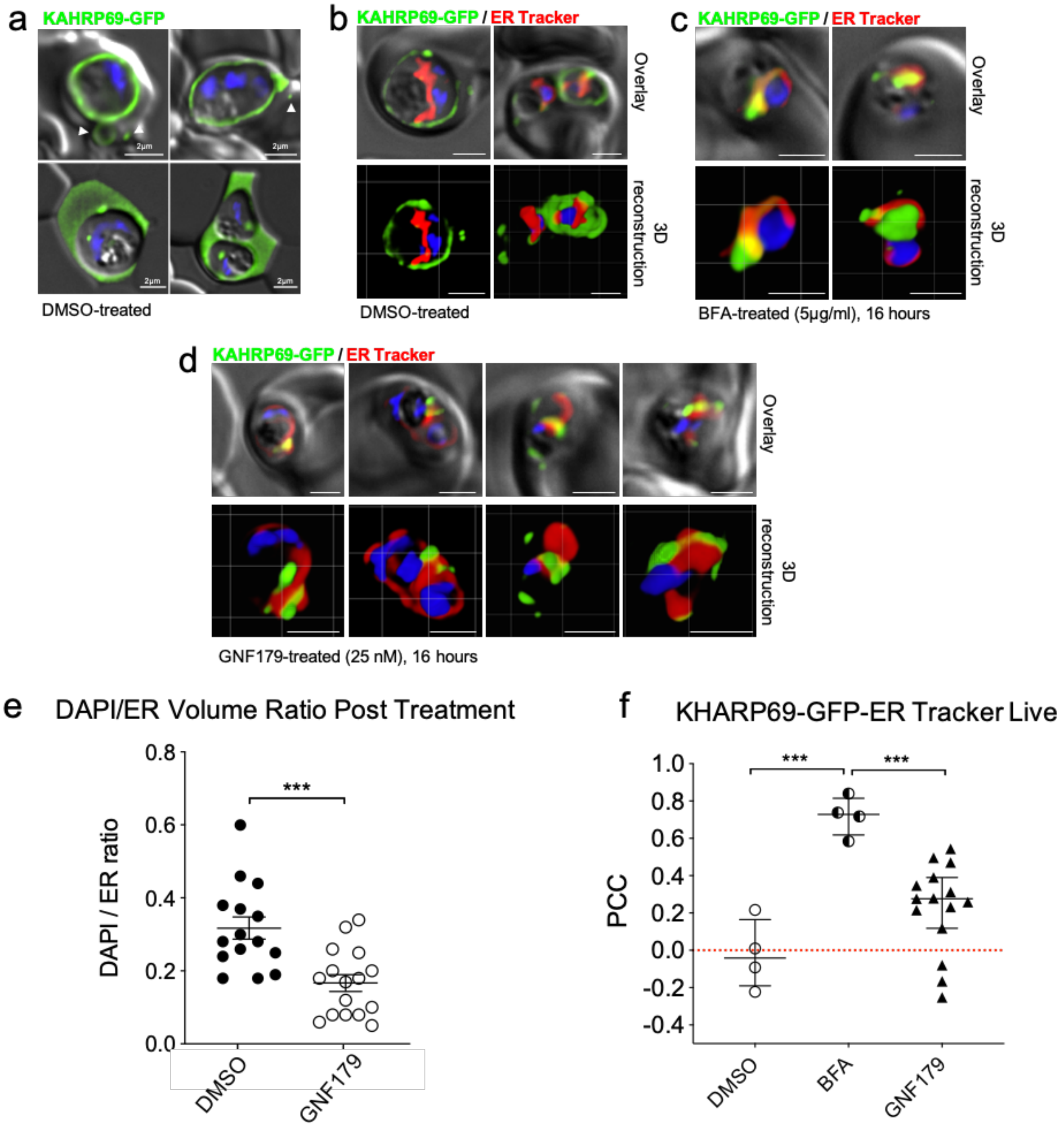

Supplementary Figure 5. GNF179 treatment increases volume of ER relative to nucleus in Dd2attB KAHRP69-GFP parasites. Red, ER Tracker; Green, GFP; Blue, DAPI. (a) Different export patterns of KAHRP69-GFP in the absence of drug treatment. (b) DMSO-treated parasites. (c) Treatment with 5 µg/ml brefeldin A for 16 hours. (d) Treatment with 25 nM GNF179 for 16 hours. (e) Quantitation of the 3D Volume ratio of DAPI to ER tracker in 12 images. (f) Quantification of colocalization between KAHRP69-GFP and ER Tracker indicated by Pearson correlation coefficient (PCC); Each dot represents an individual parasitized red blood cell. Statistical significance was determined using an unpaired, two-tailed t-test, where * is $p<0.05$; ** is $p<0.01$; *** is $p<0.001$; and ****is $p<0.0001$. Scale bars: 2 µm

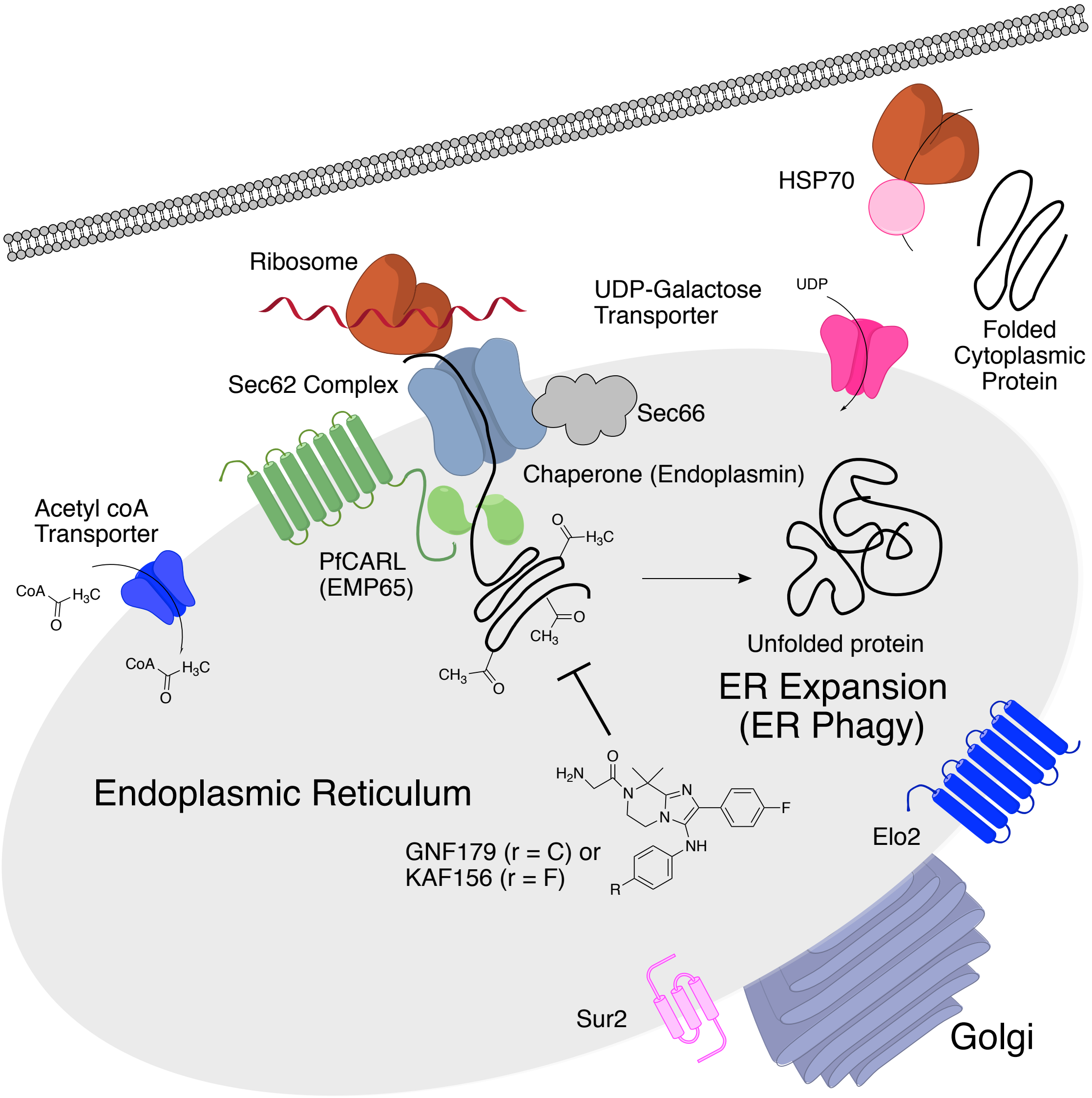

Supplementary Figure 6. Model that shows how IZP treatment could produce the sets of cellular phenotypes that are observed after treatment. In the model, IZPs inhibit the production of properly folded proteins, potentially by interfering with post-translational modification or folding. Mutations that slow the process of protein processing, as those in the Acetyl CoA transporter, *pfcarl* or *sec66* are beneficial. The accumulation of unprocessed proteins leads to ER expansion. Mutations in the autophagy and sphingolipid pathways (*elo2*, *sur2*) in yeast change the balance of ER phagy, potentially also increasing transit time and providing a slight growth advantage in the presence of GNF179 or KAF156.

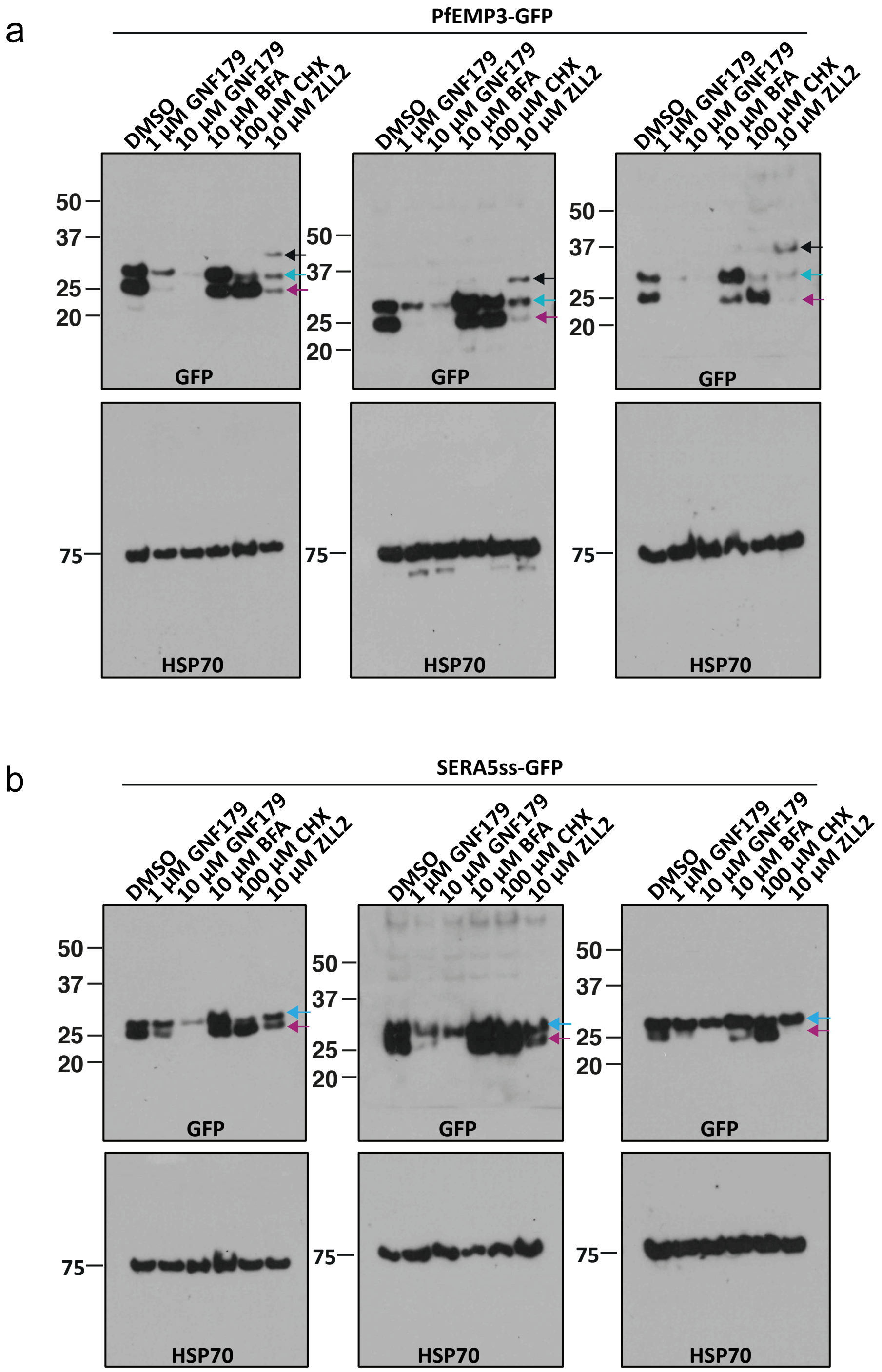

Supplementary Figure 7. Uncropped versions of western blots. Uncropped versions of three biological replicates of western blots presented in Figures 3a and 3b. (a)Protein expression levels of PfEMP3-GFP reporter. This fusion includes the signal peptide and PEXEL motif of PfEMP3. By immunoblot, 3 protein products are seen with anti-GFP antibodies. The three indicated bands, in response to probing with GFP, are as follows: 1. Full length protein (Black arrow), 2. PEXEL cleaved protein (blue arrow), 3. GFP degradation product (magenta arrow). HSP70 is used as a loading control. (b) SERA5ss-GFP fusion reporter treated with GNF179. By immunoblot with anti-GFP antibodies we see two protein products for this construct: 1. Signal peptide cleaved (blue arrow) and 2. GFP degradation product (magenta arrow). HSP70 serves as a loading control.

SUPPLEMENTARY TABLES

Clone Name	Total Base Pairs Sequenced	Mean Coverage (X)	Percent Reads Aligned to Reference	Percent Bases with 20 or More Reads	IC_{50} (μM)
**Green-Monster**	557,641,556	42.3	98.7	95.0	45.39 ± 5.7
**GNF179-G72**	1,085,965,416	59.2	99.2	94.0	63.45 ± 2.6
**GNF179-R1-2**	483,257,249	31.1	99.1	83.7	80.12 ± 14.6
**GNF179-R10-2**	868,810,757	49.1	99.4	93.4	90.31 ± 14.8
**GNF179-R12-2**	1,238,282,174	71.8	99.4	95.0	67.22 ± 10.4
**GNF179-R12h2**	504,425,192	30.9	99.4	85.3	89.65*
**GNF179-R13-2**	938,610,879	58.8	99.5	94.4	59.68 ± 11.5
**GNF179-R14-2**	719,981,126	46.4	99.7	92.0	77.83 ± 7.3
**GNF179-R18g1**	510,038,211	30.7	99.5	85.2	137.66 ± 5.1
**GNF179-R19g2**	607,361,091	35.3	99.4	89.1	109.35 ± 23.1
**GNF179-R7-2**	1,137,607,696	66.7	99.4	94.6	71.11 ± 9.5
**GNF179-R8h2**	654,463,110	38.2	99.3	90.0	138.05*
**GNF179-R9-2**	945,657,737	61.3	99.4	94.2	81.18 + 6.3
**GNF179-R9f2**	697,615,853	40.2	99.4	87.0	94.7 ± 41.3

Supplementary Table 1. Sequencing and alignment statistics for the 13 GNF179-resistant *S. cerevisiae* strains sequenced in this study. All genome sequences for the 13 IZP-resistant *S. cerevisiae* strains have been placed in the short-read sequence archive (http://www.ncbi.nlm.nih.gov/sra) under accession code STUDY: PRJNA381796 (SRP107357). *Replicate not available

Strain	Species	Genotype	Method or Ref.
ABC_{16}-Monster	Sc	*MATa adp1Δ snq2Δ ycf1Δ pdr15Δ yor1Δ vmr1Δ pdr11Δ nft1Δ bpt1Δ ybt1Δ ynr070wΔ yol075cΔ aus1Δ pdr5Δ pdr10Δ pdr12can1Δ::*GMToolkit-**a** *lyp1Δ his3Δ1 leu2Δ0 ura3Δ0 met15Δ0* (deletions for the ABC transporter genes are marked with *[tetO₂pr-GFP, URA3]*).	[1]
BY4742		*MATα his3Δ1 leu2Δ0 lys2Δ0 ura3Δ0*	
Sec66Δ	Sc	*MATα his3Δ1 leu2Δ0 lys2Δ0 ura3Δ0 sec66::KANMX4*	[2,3]
Sec72Δ	Sc	*MATα his3Δ1 leu2Δ0 lys2Δ0 ura3Δ0 sec72::KANMX4*	[2,3]
Elo2Δ	Sc	*MATα his3Δ1 leu2Δ0 lys2Δ0 ura3Δ0 elo2::KANMX4*	[2,3]
Sur2Δ	Sc	*MATα his3Δ1 leu2Δ0 lys2Δ0 ura3Δ0 sur2::KANMX4*	[2,3]
Atg15Δ	Sc	*MATα his3Δ1 leu2Δ0 lys2Δ0 ura3Δ0 atg15::KANMX4*	[2,3]
GNF179-G72	Sc	ABC_{16}-Monster	Evolved
GNF179-R1-2	Sc	ABC_{16}-Monster	Evolved
GNF179-R10-2	Sc	ABC_{16}-Monster	Evolved
GNF179-R12-2	Sc	ABC_{16}-Monster	Evolved
GNF179-R12h2	Sc	ABC_{16}-Monster	Evolved
GNF179-R13-2	Sc	ABC_{16}-Monster	Evolved
GNF179-R14-2	Sc	ABC_{16}-Monster	Evolved
GNF179-R18g1	Sc	ABC_{16}-Monster	Evolved
GNF179-R19g2	Sc	ABC_{16}-Monster	Evolved
GNF179-R7-2	Sc	ABC_{16}-Monster	Evolved
GNF179-R8h2	Sc	ABC_{16}-Monster	Evolved
GNF179-R9-2	Sc	ABC_{16}-Monster	Evolved
GNF179-R9f2	Sc	ABC_{16}-Monster	Evolved
EAW289	Sc	ABC_{16}-Monster	Edited
EAW327	Sc	ABC_{16}-Monster	Edited
EAW361	Sc	ABC_{16}-Monster	Edited
EAW277	Sc	ABC_{16}-Monster	Edited
3D7	Pf		[4]
Dd2	Pf	*Pfcrt* (K76T), *pfmdr1* (CNV)	[5]
KAD452-R3	Pf	Dd2 *pfcarl* (M81I, L830V and S1076I)	Evolved
Dd2-ACTStop	Pf	Dd2 *pfact (S242*)*	Edited [6]
EMP3-GFP	Pf	*Pfemp3* (residues 1-82) fused with GFP	[7]
SERA5ss-GFP	Pf	*Pfsera5* with signal peptide (residues 1–25) fused with GFP	[8]
Dd2attB KAHRP69-GFP	Pf	Dd2 *attB::KAHRP69-GFP*	Recombinant
Sec62 HA-Glcn	Pf	HA-*glmS* tagged *pfsec62*	[9]

Supplementary Table 2. Strains used in this study. Sc, *S. cerevisiae*, Pf, *P. falciparum*.

Name	Oligonucleotide Sequence
gRNA Forward Primer Elo2 G183C	tcATAATAAGGCAGTAGCGCCAgTTTTAGAGCTAGAAATAGCAAG
gRNA Reverse Primer Elo2 G183C	aacTGGCGCTACTGCCTTATTATgATCATTTATCTTTCACTGCGGAG
Donor Template Sequence Elo2 G183C	TGCTAAAACATAAAAAATTGACATTTTTGCATACTTATCACCATTGCG CTACTGCCTTATTATGTTACACCCAATTGATGGGCACCACA
Sequencing Primer Elo2 G183C Coding Strand	GCACGGGTTATACTTCGCTATC
Sequencing Primer Elo2 G183C Non-Coding Strand	CTGGCAGCCAAGAAATAGTACC
gRNA Forward Primer Elo3 Y307*	tcATCCAATCTTACAAGAAAGGgTTTTAGAGCTAGAAATAGCAAG
gRNA Reverse Primer Elo3 Y307*	aacCCTTTCTTGTAAGATTGGATgATCATTTATCTTTCACTGCGGAG
Donor Template Sequence Elo3 Y307*	ACATCTTATTTGCTTTTGTTTATTTCCTTCTACATCCAATCTTAAAAGA AAGGTGGTAAAAAGACAGTCAAGAAGGAATCTGAAGTTTCC
Sequencing Primer Elo3 Y307* Coding Strand	TGTTATGGTACTCAGGCTGCTG
Sequencing Primer Elo3 Y307* Non-Coding Strand	CCTGGAAGAGACCTTGGTGTTA
gRNA Forward Primer Sec66 M1 read through	tcTTCATTAAATTCGGACATATgTTTTAGAGCTAGAAATAGCAAG
gRNA Reverse Primer Sec66 M1 read through	aacATATGTCCGAATTTAATGAAgATCATTTATCTTTCACTGCGGAG
Donor Template Sequence Sec66 M1 read through	AGTACAGGAAAGAGGTACGCACAACTACTTGAGTTTGCCAATATTTC CGAATTTAATGAAACAAAATTCTCCAACAACGGGACGTTTTTT
Sequencing Primer Sec66 M1 read through Coding Strand	GTAGACGCATCTTATTACCCGC
Sequencing Primer Sec66 M1 read through Non-Coding Strand	TGAGTGGGGTATAAACGGAGAT
gRNA Forward Primer Sec66 S107*	tcATCATTAAAGTTAAAAGAGTgTTTTAGAGCTAGAAATAGCAAG
gRNA Reverse Primer Sec66 S107*	aacACTCTTTTAACTTTAATGATgATCATTTATCTTTCACTGCGGAG
Donor Template Sequence Sec66 S107*	GAAGGCCGCTTTATTGAACAGAGGAGCAGAGTCTGTTAGACGATGAT TAAAGTTAAAAGAGTTGGCTCCTCAGATAAACCTTCTATATAA
Sequencing Primer Sec66 S107* Coding Strand	CATCCATATTTGACGAAAACGA
Sequencing Primer Sec66 S107* Non-Coding Strand	GGTTGCAATCTTTCAGCTTCTT

Supplementary Table 3. Oligonucleotides used in this study.

Gene Name	Parent strain/Clone Name	Amino Acid Change	GNF179 IC_{50} (nM) (95% CI)	GNF179 Fold resistance (FR)	Reference
Pfcarl Cyclic amine resistance transporter	Dd2/Mutant H5	P822L	274.4 (201.7–373.3)	43	[10]
	Dd2/Mutant D6	S1076I	141.6 (125.2–160.1)	22	[10]
	Dd2/Mutant H8	S1076I	185.6 (155.8–221.2)	29	[10]
	Dd2/S1-179-Dd2-2B	E834K	153.8 (137.7–171.6)	24	[10]
	Dd2/KAD452-R3	M81I, L830V and S1076I	1020 $\pm$ 120	340	[11]
Pfugt UDP-galactose transporter	Dd2/Mutant A3	F37V	2410 (1816–3198)	382	[10]
	Dd2/Mutant A9	F37V	3074 (2749–3436)	487	[10]
	Dd2/Mutant B6	F37V	3045 (2738–3387)	483	[10]
	Dd2/Mutant A2	F37V	2858 (1936–4219)	453	[10]
	Dd2/Mutant B5	F37V	2660 (2099–3371)	422	[10]
	Dd2/Mutant D8	F37V	2210 (1710–2855)	350	[10]
	Dd2/Mutant E8	F37V	3402 (2174–5324)	539	[10]
Pfact Acetyl-CoA transporter	3D7/S2-179-3D7-1B	Intronic	7962 (4152–15269)	2568	[10]
	3D7/S2-179-3D7-2A	Intronic	895.7 (316.2–2537)	289	[10]
	3D7/S2-179-3D7-2C	Intronic	3633 (2209–5975)	1172	[10]
	3D7/S2-179-3D7-3A	L253*	1613 (1121–2322)	520	[10]
	3D7/S2-179-3D7-3C	L253*	5185 (4076–6596)	1	[10]
	3D7/S1-179-3D7-1B	A94T	868.6 (534.4–1412)	280	[10]
	3D7/S1-179-3D7-2A	D165N	693.4 (451.1–1066)	224	[10]
	3D7/S1-179-3D7-3A	S110R	285 (130.7–619.9)	92	[10]
	3D7/S1-179-3D7-3B	S110R	3566 (3119–4076)	1150	[10]
	Dd2/Dd2_Mutant B3	S242*	2230 (1556–3196)	353	[10]
	Dd2/S1-179-Dd2-1A	C193*	1898 (1492–2416)	301	[10]
	Dd2/S1-179-Dd2-1B	G559R	1091 (854.7–1392)	173	[10]
	Dd2/S1-179-Dd2-3A	R108K	3141 (2594–3803)	499	[10]

Supplementary Table 4. Mutations identified from evolved GNF179-resistant *P. falciparum* lines. 72 hrs IC_{50}s against GNF179 and fold resistances (calculated relative to the parental strain) are indicated.

SUPPLEMENTARY METHODS

Conjugation of Coumarin-1 and NBD with GNF179

GNF179 was conjugated with a coumarin-1 fluorophore as previously described[12]. Briefly, Meldrum's acid was acylated with methyl 5-chloro-5-oxovalerate and subsequently treated with methanol to provide coumarin β-keto ester. Next, the β-keto ester was first reacted with resorcinol under acidic conditions and then hydrolyzed with lithium hydroxide to provide 4-(7-hydroxy-2-oxo-2H-chromen-4-yl) coumarin butanoic acid 2.8 Finally, GNF179 and coumarin butanoic acid was coupled under standard EDCI/DMAP coupling conditions to yield the probe Coumarin-1-GNF179. To construct the NBD modified version, GNF179 was conjugated with a nitrobenzoxadiazole (NBD) fluorescent label by reacting GNF179, triethylamine, and commercially available NBD-Cl in dimethylformamide.

All reactions were performed in flame- or oven-dried glassware sealed with rubber septa and under nitrogen atmosphere, unless otherwise indicated. Air- and/or moisture-sensitive liquids or solutions were transferred by cannula or syringe. Organic solutions were concentrated by rotary evaporator at 30 millibar with the water bath heated to not more than 50°C, unless specified otherwise. Thin-layer chromatography (TLC) was performed using 0.2 mm commercial silica gel plates (silica gel 60, F254, EMD Chemicals). Nuclear Magnetic Resonance (NMR) spectra were recorded on a Varian (^{1}H NMR: $CDCl_3$ (7.26) at 600 MHz; ^{13}C NMR: $CDCl_3$ (77.16) at 151 MHz). All spectra were taken in $CDCl_3$ with shifts reported in parts per million (ppm) referenced to protium or carbon of the solvent (7.26 or 77.16, respectively). Coupling constants are reported in Hertz (Hz). Data for ^{1}H-NMR are reported as follows: chemical shift (ppm, reference to protium; s = single, d = doublet, t = triplet, q = quartet, dd = doublet of doublets, m = multiplet, coupling constant (Hz), and integration). High Resolution Mass Spectra (HRMS) were acquired on an Agilent 6230 High Resolution time-of-flight mass spectrometer and reported as m/z for the molecular ion [M+H]+.

GNF179-1-Coumarin

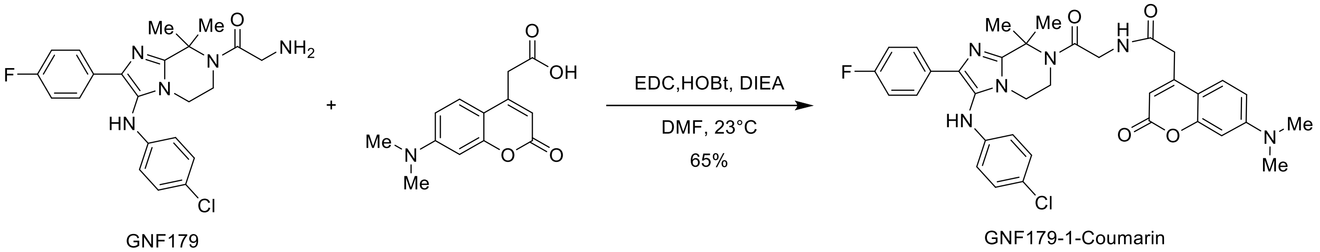

GNF179-1-Coumarin: A dry 5 mL round-bottom flask equipped with a stir bar, sealed with a rubber septum was charged with 7-dimethylaminocoumarin-4-acetic acid (4 mg, 0.016 mmol, 1.0 equiv.) and GNF179 (7 mg, 0.016 mmol, 1.0 equiv.) in dry DMF (0.2 mL). The solution was cooled to 5 °C and solid EDC (4 mg, 0.023 mmol, 1.42 equiv.), HOBt (3 mg, 0.019 mmol, 1.2 equiv.) and neat DIEA (3 µL, 0.019 mmol, 1.2 equiv.) were added to the mixture. The solution as stirred for 30 mins and the reaction was allowed to warm to ambient temperature and react for 18 hours. The reaction was then diluted with EtOAc (3 mL) and washed with water (1 mL). The mixture was extracted with EtOAc (2 mL). The combined organic layers were dried over sodium sulfate, filtered, and concentrated. The crude product was purified by silica column chromatography, eluting with Hexanes: EtOAc (10: 90) affording the title compound (6 mg, 9.13 mmol, 63%) as yellow color solid.

NMR results: $\mathbf{R_f}$ = 0.5 (silica gel, 100 EtOAc); **^{1}H NMR** (600 MHz, $CDCl_3$) δ 7.73 (dd, *J* = 8.5, 5.6 Hz, 2H), 7.40 (d, *J* = 9.0 Hz, 1H), 7.13 (d, *J* = 8.6 Hz, 2H), 6.95 (m, 3H), 6.56 (m, 3H), 6.35 (d, *J* = 2.1 Hz, 1H), 6.01 (s, 1H), 5.79 (s, 1H), 4.06 (d, *J* = 3.7 Hz, 2H), 3.68 – 3.64 (m, 2H), 3.63 (s, 2H), 3.47 (m, 2H), 3.00 (s, 6H), 1.88 (s, 6H). **^{13}C NMR** (151 MHz, $CDCl_3$) δ 167.98, 167.27, 162.70, 162.02, 161.07, 155.94, 153.11, 149.83, 147.44, 144.32, 133.72, 129.74, 127.64, 127.59, 125.60, 124.46, 122.96, 115.50, 115.36, 114.57, 110.15, 109.20, 108.41, 98.12, 60.34, 43.51, 41.69, 41.01, 40.17, 39.89, 26.47. **HRMS**: *m/z*: calcd for $C_{35}H_{35}ClFN_6O_4$: 657.2387; found 657.2384 $[M + H]^+$.

GNF179-NBD

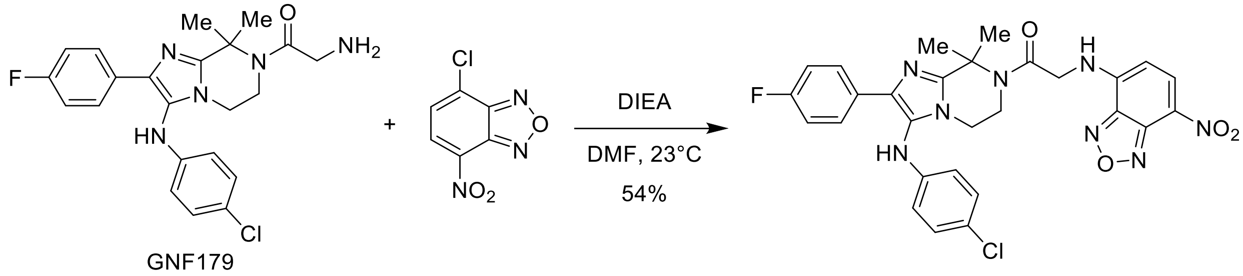

GNF179-NBD: To a stirred solution of GNF179 (6 mg, 0.014 mmol, 1.0 equiv.) and 4-chloro-7-nitrobenzofuran (3 mg, 0.014 mmol, 1.0 equiv.) in dry DMF (0.2 mL) was added neat DIEA (4 µL, 0.021 mmol, 1.5 equiv.). The solution was stirred for 16 hours at 23 °C. The reaction was then diluted with EtOAc (2.5 mL) and water (1.2 mL) and the organic layer was collected. The mixture was extracted with additional EtOAc (2 mL). The EtOAc layers were dried over sodium sulfate, filtered, and concentrated. The crude product was purified by silica column chromatography, eluting with Hexanes: EtOAc (40: 60) afforded the title compound (4 mg, 6.77 mmol, 52%) as light brown solid.

NMR results: $\mathbf{R_f}$ = 0.6 (silica gel, 30:70 Hexanes: EtOAc); **^{1}H NMR** (600 MHz, $CDCl_3$) δ 8.35 (d, *J* = 8.4 Hz, 1H), 7.72 – 7.65 (m, 2H), 7.59 (s, 1H), 7.13 (d, *J* = 8.2 Hz, 2H), 6.94 (t, *J* = 8.3 Hz, 2H), 6.57 (d, *J* = 8.2 Hz, 2H), 6.13 (s, 1H), 5.60 (s, 1H), 4.30 (s, 2H), 3.91 (m, 2H), 3.80 (m, 2H), 2.00 (s, 6H). **^{13}C NMR** (151 MHz, $CDCl_3$) δ 162.72, 161.08, 147.27, 144.22, 133.82, 129.81, 129.65, 129.64, 127.67, 127.62, 124.62, 122.93, 115.53, 115.38, 114.59, 60.93, 56.09, 41.95, 41.03, 29.80, 26.52. **HRMS**: *m/z*: calcd for $C_{28}H_{25}ClFN_8O_4$: 591.1666; found 591.1662 $[M + H]^+$.

The potency of both GNF179 fluorescent conjugate was assessed via a 72-hour SYBR Green I fluorescence IC_{50} assay[13].

SUPPLEMENTARY REFERENCES

1. Suzuki, Y., *et al.* The green monster process for the generation of yeast strains carrying multiple gene deletions. *J Vis Exp*, e4072 (2012).
2. Winzeler, E.A., *et al.* Functional characterization of the S. cerevisiae genome by gene deletion and parallel analysis. *Science* **285**, 901-906 (1999).
3. Giaever, G., *et al.* Functional profiling of the Saccharomyces cerevisiae genome. *Nature* **418**, 387-391 (2002).
4. Walliker, D., *et al.* Genetic analysis of the human malaria parasite Plasmodium falciparum. *Science* **236**, 1661-1666 (1987).
5. Guinet, F., *et al.* A developmental defect in Plasmodium falciparum male gametogenesis. *J Cell Biol* **135**, 269-278 (1996).
6. LaMonte, G., *et al.* Mutations in the Plasmodium falciparum Cyclic Amine Resistance Locus (PfCARL) Confer Multidrug Resistance. *MBio* **7**(2016).
7. Boddey, J.A., *et al.* An aspartyl protease directs malaria effector proteins to the host cell. *Nature* **463**, 627-631 (2010).
8. Sleebs, B.E., *et al.* Inhibition of Plasmepsin V Activity Demonstrates Its Essential Role in Protein Export, PfEMP1 Display, and Survival of Malaria Parasites. *PLoS Biol* **12**(2014).
9. Marapana, D.S., *et al.* Plasmepsin V cleaves malaria effector proteins in a distinct endoplasmic reticulum translocation interactome for export to the erythrocyte. *Nat Microbiol* **3**, 1010-1022 (2018).
10. Lim, M.Y., *et al.* UDP-galactose and acetyl-CoA transporters as Plasmodium multidrug resistance genes. *Nat Microbiol* **1**, 16166 (2016).
11. Magistrado, P.A., *et al.* Plasmodium falciparum Cyclic Amine Resistance Locus (PfCARL), a Resistance Mechanism for Two Distinct Compound Classes. *ACS Infect Dis* **2**, 816-826 (2016).
12. Alexander, M.D., *et al.* A central strategy for converting natural products into fluorescent probes. *Chembiochem* **7**, 409-416 (2006).
13. Johnson, J.D., *et al.* Assessment and continued validation of the malaria SYBR green I-based fluorescence assay for use in malaria drug screening. *Antimicrob Agents Chemother* **51**, 1926-1933 (2007).
