# Supplementary material for: Pan-active imidazolopiperazine antimalarials target the Plasmodium falciparum intracellular secretory pathway
Source: Nat Commun. 2020 Apr 14;11:1780. doi: 10.1038/s41467-020-15440-4 (PMC7156427; doi:10.1038/s41467-020-15440-4)
Supplement: Supplementary file 2 — Reporting Summary [file 41467_2020_15440_MOESM2_ESM.pdf]

nature research

Corresponding author(s): Elizabeth Winzeler

Last updated by author(s): Feb 1, 2020

Reporting Summary

Nature Research wishes to improve the reproducibility of the work that we publish. This form provides structure for consistency and transparency in reporting. For further information on Nature Research policies, see Authors & Referees and the Editorial Policy Checklist .

Statistics

For all statistical analyses, confirm that the following items are present in the figure legend, table legend, main text, or Methods section.

n/a	Confirmed	
☐	☒	The exact sample size (*n*) for each experimental group/condition, given as a discrete number and unit of measurement
☐	☒	A statement on whether measurements were taken from distinct samples or whether the same sample was measured repeatedly
☐	☒	The statistical test(s) used AND whether they are one- or two-sided<br>*Only common tests should be described solely by name; describe more complex techniques in the Methods section.*
☒	☐	A description of all covariates tested
☒	☐	A description of any assumptions or corrections, such as tests of normality and adjustment for multiple comparisons
☐	☒	A full description of the statistical parameters including central tendency (e.g. means) or other basic estimates (e.g. regression coefficient) AND variation (e.g. standard deviation) or associated estimates of uncertainty (e.g. confidence intervals)
☐	☒	For null hypothesis testing, the test statistic (e.g. F, t, r) with confidence intervals, effect sizes, degrees of freedom and P value noted<br>*Give P values as exact values whenever suitable.*
☒	☐	For Bayesian analysis, information on the choice of priors and Markov chain Monte Carlo settings
☒	☐	For hierarchical and complex designs, identification of the appropriate level for tests and full reporting of outcomes
☒	☐	Estimates of effect sizes (e.g. Cohen's *d*, Pearson's *r*), indicating how they were calculated

Our web collection on statistics for biologists contains articles on many of the points above.

Software and code

Policy information about availability of computer code

Data collection	No software was used to collect data in this study
Data analysis	Yeast Genome Sequencing<br>DNA sequencing was done using Illumina HiSeq 2500 in RapidRun mode to generate paired-end reads 100bp in length. Resulting reads were then aligned to the S. cerevisiae 288C reference genome (assembly R64) using BWA-mem and further processed using Picard Tools (http://broadinstitute.github.io/picard/). SNVs and INDELs were called using GATK HaplotypeCaller, filtered based on GATK recommendations and annotated with SnpEff.<br><br>Yeast Functional Genetics Analysis<br>Gene maps were generated using Illustrator of Biological Sequences (IBS) software package, while Protein-Protein Interaction (PPI) networks were generated using the STRING database.<br><br>Evaluation of Drug Sensitivity (IC50)<br>GraphPad Prism (commercially available software) was used to plot dose-response curves and calculate IC50s for all datasets<br><br>Sphingolipid Analysis<br>Sphingolipid quantitation was performed using Agilent Quantitative Analysis Software and compared using GraphPad Prism

For manuscripts utilizing custom algorithms or software that are central to the research but not yet described in published literature, software must be made available to editors/reviewers. We strongly encourage code deposition in a community repository (e.g. GitHub). See the Nature Research guidelines for submitting code & software for further information.

Data

Policy information about availability of data

All manuscripts must include a data availability statement. This statement should provide the following information, where applicable:

- Accession codes, unique identifiers, or web links for publicly available datasets
- A list of figures that have associated raw data
- A description of any restrictions on data availability

All genome sequences for the 13 IZP-resistant S. cerevisiae strains have been placed in the short-read sequence archive (http://www.ncbi.nlm.nih.gov/sra) under accession code STUDY: PRJNA381796 (SRP107357). Whole-genome sequences for KAD452-R3 can be downloaded from (located on NAS server: Victoria, collaborative sequencing projects, Lamonte_KAF156R). The authors declare that all other data supporting the findings of this study are available within the supplementary files, or from the authors upon request.

Field-specific reporting

Please select the one below that is the best fit for your research. If you are not sure, read the appropriate sections before making your selection.

[x] Life sciences [ ] Behavioural & social sciences [ ] Ecological, evolutionary & environmental sciences

For a reference copy of the document with all sections, see nature.com/documents/nr-reporting-summary-flat.pdf

Life sciences study design

All studies must disclose on these points even when the disclosure is negative.

Sample size	No sample size calculation was performed as it was deemed unnecessary for the study design.
Data exclusions	No data was excluded from presented analyses
Replication	Findings presented in this study are reproduced across all replicates attempted.
Randomization	Randomization was deemed unnecessary for the study design and the relatively small number of replicates for experimental conditions tested.
Blinding	Blinding was not possible as samples coming to different cell lineages (resistant vs. non-resistant) or treatment conditions (treated with drug A, B, C or sham-treated) were cultivated for multiple generations, and harvested side by side.

Reporting for specific materials, systems and methods

We require information from authors about some types of materials, experimental systems and methods used in many studies. Here, indicate whether each material, system or method listed is relevant to your study. If you are not sure if a list item applies to your research, read the appropriate section before selecting a response.

Materials & experimental systems

n/a	Involved in the study	
[ ]	[x]	Antibodies
[ ]	[x]	Eukaryotic cell lines
[x]	[ ]	Palaeontology
[x]	[ ]	Animals and other organisms
[x]	[ ]	Human research participants
[x]	[ ]	Clinical data

Methods

n/a	Involved in the study	
[x]	[ ]	ChIP-seq
[x]	[ ]	Flow cytometry
[x]	[ ]	MRI-based neuroimaging

Antibodies

Antibodies used

Primary Antibodies
Commercial Anti-GFP, Roche, Cat no.: 11814460001, Clone name: mouse IgG1κ (clones 7.1 and 13.1)
Commercial Anti-GFP: Living Colors® Full-Length GFP Polyclonal Antibody, Takara (Clontech), Cat. No. 632592
Commercial Anti-Ubiquitin, Cell Signalling Technology, Cat no.: 3936T, Clone name: PD41, Lot Number: 03/2018
Commercial Anti-PDI, Enzo Life Sciences, Cat.No. ADI-SPA-891-D, clone 1D3
Commercial Anti-eIF2a-p (Cell Signaling Technology-119A11)
Anti-ERD2 was purchased from MR4: MRA-1 Polyclonal Anti-Plasmodium falciparum PfERD2 (antiserum, Rabbit) (Polyclonal Antiserum)
Rabbit anti-SERA5 antibodies were originally generated from a previous study PMID: 12228245

Rabbit anti-PTP2 and anti-PIESA2 (PIESP2), antibodies were originally generated from a previous study PMID: 1861401
Rabbit anti-HSP70 antibodies were originally generated in a previous study Bianco et al., PNAS 1986 (https://doi.org/10.1016/j.cell.2008.04.051)

Secondary Antibodies
Commercial Goat anti-Mouse IgG1 Cross-Adsorbed Secondary Antibody, Alexa Fluor 488, Thermo Fisher, Cat. No. A-21121
Commercial Goat anti-Rabbit IgG (H+L) Cross-Adsorbed Secondary Antibody, Alexa Fluor 568, Thermo Fisher, Cat. No. A-11011
Commercial Goat anti-Rat IgG (H+L) Cross-Adsorbed Secondary Antibody, Alexa Fluor 647, Thermo Fisher, Cat. No. A-21247

Validation

All commercially available antibodies were validated by the respective manufacturers.
Anti-ERD2 was validated in a previous publication PMID:8223485
Rabbit anti-SERA5 was validated in this publication by western blot.
Rabbit anti-PTP2 and anti-PIESA2 (PIESP2), antibodies were and validated in this publication by western blot and subcellular localisation.
Rabbit anti-HSP70 antibodies were validated in this publication by western blot.

Eukaryotic cell lines

Policy information about cell lines

Cell line source(s)

Yeast Lines
Green Monster lines were acquired through an MTA from the laboratory of Dr. FP Roth. The genetic modified cell line was originally generated as a part of a previous study PMID: 21217751

Wild-type Parasite Lines
WT Plasmodium falciparum lines are routinely used, lab-adapted strains that were originally acquired from the Malaria Research and Reference Reagent Resource Center (MR4) - BEI Resources

Non-wild-type Parasite Lines
The PfEMP3-GFP strain was generated from a previous study PMID: 20130643
The SERA5ss-GFP strain was generated from a previous study PMID: 24983235
the Sec62-HA-glmS strain was generated from a previous study PMID: 30127496
The The KAHRP69-GFP reporter line was generated on a parasite background belonging to a Dd2attB parasite line that was originally generated from a previous study PMID: 16862136
The KAD452-R3 was generated through in vitro evolution of a Dd2 background line, and initially reported in a previous study PMID: 24913172
The Dd2-ACT* line was generated from a previous study PMID: 27642791

Authentication

Green Monster and wild-type parasite lines are authenticated by virtue of being continually used for in vitro drug selection, and periodically being subjected to whole genome sequencing which involves mapping to the reference Sc or Pf genome, respectively.

Mycoplasma contamination

Wild-type parasite lines have been periodically tested, and are confirmed to be negative for Mycoplasma contamination.

Commonly misidentified lines
(See ICLAC register)

No commonly misidentified cell lines were used in this study.
