# Supplementary material for: Pan-active imidazolopiperazine antimalarials target the Plasmodium falciparum intracellular secretory pathway
Source: Nat Commun. 2020 Apr 14;11:1780. doi: 10.1038/s41467-020-15440-4 (PMC7156427; doi:10.1038/s41467-020-15440-4)
Supplement: Supplementary file 3 — Description of Additional Supplementary Files [file 41467_2020_15440_MOESM3_ESM.pdf]

Description of Additional Supplementary Files

File Name: Supplementary Data 1

Description: **Complete set of mutations observed in S. cerevisiae in vitro resistance evolutions. (Attached Excel Table)** Clone #: The numerical designation for each S. cerevisiae GNF179 resistant clone in this study. Chr #: Chromosome # which harbors the identified mutations.
